# lncRNA MAGI2-AS3 Exerts Antioncogenic Roles in Hepatocellular Carcinoma via Regulating the miR-519c-3p/TXNIP Axis

**DOI:** 10.1155/2021/5547345

**Published:** 2021-08-27

**Authors:** Huamei Wei, Qianli Tang, Anmin Wang, Ya Zhang, Zebang Qin, Wenchuan Li, Zuoming Xu, Jianchu Wang, Jian Pu

**Affiliations:** ^1^Department of Pathology, Affiliated Hospital of Youjiang Medical University for Nationalities, Baise, Guangxi 533000, China; ^2^Department of Hepatobiliary Surgery, Affiliated Hospital of Youjiang Medical University for Nationalities, Baise, Guangxi 533000, China; ^3^Graduate College, Youjiang Medical University for Nationalities, Baise, Guangxi 533000, China

## Abstract

**Introduction:**

Our work was aimed to explore the mechanisms of MAGI2 antisense RNA 3 (MAGI2-AS3) in regulating hepatocellular carcinoma (HCC) carcinogenesis.

**Methods:**

MAGI2-AS3, microRNA-519c-3p (miR-519c-3p), and thioredoxin interacting protein (TXNIP) levels in HCC were detected by the RT-qPCR method. Cell proliferation and apoptosis rate were measured using Cell Counting Kit-8 assay and flow cytometry assay. Relationship between MAGI2-AS3, TXNIP, and miR-519c-3p were analyzed via luciferase activity assay, RNA pull-down assay, and RNA immunoprecipitation assay. Mouse xenograft models of HCC were conducted to explore the roles of MAGI2-AS3 in vivo.

**Results:**

MAGI2-AS3 levels were elevated, and miR-519c-3p decreased in HCC. MAGI2-AS3 overexpression inhibits while its knockdown stimulates HCC cell growth through miR-519c-3p. Moreover, miR-519c-3p overexpression stimulates HCC cell growth. MAGI2-AS3 serves as competing endogenous RNA (ceRNA) of miR-519c-3p to regulate TXNIP in HCC. And, TXNIP upregulation weakened the influence of MAGI2-AS3 knockdown on HCC cell behaviors. Additionally, MAGI2-AS3 overexpression suppressed HCC tumor growth in vivo.

**Conclusion:**

MAGI2-AS3 inhibits HCC tumorigenesis through miR-519c-3p/TXNIP axis in vitro and in vivo, indicating MAGI2-AS3 plays a crucial role in HCC development.

## 1. Introduction

In recent years, the numbers of newly diagnosed and death cases for liver cancer have gradually increased and becomes a cancer type with highest mortality worldwide [1]. Although the improvements are on treatment methods, the prognosis of liver cancer remains poor due to most patients are diagnosed at advanced stages [2]. Hepatocellular carcinoma (HCC) accounts for 75–85% of all liver cancer cases all over the world [3]. Hence, it is essential to discover molecular markers behind HCC development.

RNA can be generally divided into two groups, protein coding and noncoding RNAs, based on whether or not they have the ability to code proteins. Noncoding RNAs (ncRNAs) represents about 98% of all genome transcripts and can be classified into short ncRNAs (<200 nucleotides) and long ncRNAs (lncRNA, >200 nucleotides) [4]. Although only some of these lncRNAs have been functionally characterized, they are found to contribute the malignant behaviors of cancer cells and therefore could not be merely regarded as transcriptional “noise” [5].

MAGI2 antisense RNA 3 (MAGI2-AS3) is a lncRNA with dual roles, tumor suppressive role and oncogenic role, in cancers. For instance, MAGI2-AS3 was a recently found decreased expression in HCC and could inhibit HCC cell growth and metastasis via recruiting KDM1A to enhance the H3K4me2 demethylation status at the promoter region of RACGAP1 [6]. On the contrary, MAGI2-AS3 was found elevated expression in colorectal cancer and drives cancer tumorigenesis in vitro and in vivo through regulating the miR-3163/TMEM106B axis [7]. However, the roles of MAGI2-AS3 in HCC remain to be further explored.

Mechanistically, lncRNA serves as competitive endogenous RNA (ceRNA) for miRNA to modulate messenger RNA (mRNA) expression [8]. In this work, we focused on miR-519c-3p which has been revealed that could contribute to HCC growth and metastasis in vitro and in vivo by targeting B cell translocation gene 3 [9]. However, the mechanisms regarding the upregulation status of miR-519c-3p in HCC remain to be explored.

Here, we hypothesized MAGI2-AS3 may function as ceRNA for miR-519c-3p in HCC. The expression levels of MAGI2-AS3 and miR-519c-3p in HCC were analyzed. Moreover, gain and loss-of-function studies were performed to investigate the associated mechanisms of MAGI2-AS3 in HCC.

## 2. Materials and Methods

### 2.1. Patients Samples

HCC tumor tissues and adjacent noncancerous tissues were collected from 27 patients underwent treatment at Affiliated Hospital of Youjiang Medical University for Nationalities with a protocol approved by our hospital. These tissues were all stored at −80°C refrigerator until usage. The written informed consent was obtained from all participants.

### 2.2. Cell Lines and Culture

Dulbecco's modified Eagle medium (DMEM) and 10% fetal bovine serum (Invitrogen, Carlsbad, CA, USA) were used to incubate HCC cells (Huh7, Hep3B, SNU-182) and normal liver cell (THLE-3) obtained from ATCC. The incubation condition was maintained at 37°C with 5% of CO_2_.

### 2.3. Transfection

To overexpress MAGI2-AS3 or thioredoxin interacting protein (TXNIP), their full-length sequences were inserted into pcDNA3.1. To overexpress miR-519c-3p, miR-519c-3p mimic and corresponding control (miR-con) were bought from GeneChem (Shanghai, China). Tu suppress MAGI2-AS3 expression, and small interfering RNA si-MAGI2-AS3 and siR-con were purchased at GeneChem. Cells were seeded into 6-well plate and transfected with Lipo 2000 (Invitrogen) according to the manufacturer's instructions

### 2.4. RNA Extraction and Real-Time Quantitative PCR (RT-qPCR) Assay

To isolate RNA from tissues and cells, TRIzol reagent (Invitrogen) was used. Afterward, RNA sample extracted was reverse transcribed into complementary DNA using TIANScript Kit (Tiangen, Beijing, China). RT-qPCR was conducted on BAI 7500 (Applied Biosystems, Foster City, CA, USA) using SYBR Green (Takara, Dalian, China). Primers used were synthesized by GeneChem and listed as follows: MAGI2-AS3: forward, 5′‐CACCTTGCTTGACAAC‐TTGA‐3′, reverse, 5′‐CATTACAGCTCGGCTACTGC‐3′; TXNIP: forward, 5′-CGCCTCCTGCTTGAAACTA‐AC-3′, reverse, 5′-AATATACGCCGCTGGTTACACT-3′; miR-519c-3p: forward, 5′-GGCGGGAAAGTGCATCTTTTT-3′, reverse, 5′- GTCGTATCCAGTGCAGGGTCCGAGGTATTCGCACTGGATACGACATCCTC-3′; GAPDH: forward, 5′-CGCTCTCTGCTCCTCCTGTTC-3, reverse, 5′-ATCCGTTGACTCCGACCTTCAC-3′; and U6 snRNA: forward, 5′-CTCGCTTCGGCAGCACA-3′, reverse, 5′-AACGCTTCACGAATTTGCGT-3′. Expression levels were calculated with the 2-ΔΔCT method with GAPDH or U6 snRNA as endogenous control.

### 2.5. Cell Proliferation Analysis

Cell Counting Kit-8 (CCK-8; Beyotime, Haimen, China) assay was conducted to measure cell proliferation. Cells were seeded in 96-well plate and measured every 24 h using a microplate reader after the addition of CCK-8 reagent for 4 times. Optical density was set as 450 nm.

### 2.6. Cell Apoptosis Assay

Annexin V-FITC and PI were used to stain HCC cells based on manufacturer's instructions. Then, cell apoptosis rate including early to late apoptosis was analyzed at FACScan flow cytometry (BD Biosciences).

### 2.7. Bioinformatics Analysis

We explored the possible targets for MAGI2-AS3 using ENCORI and revealed miR-519c-3p was a target. In addition, TargetScan was used to explore the targets for miR-519c-3p and identified YXNIP as a possible target.

Luciferase reporter assay: sequences of MAGI2-AS3 and TXNIP containing the binding region for miR-519c-3p were inserted into pmirGLO (Promega, Madison, WI, USA) to obtain MAGI2-AS3-WT and TXNIP-WT. The site-directed mutagenesis kit (Takara) was used to generate mutant luciferase vectors: MAGI2-AS3-MT and TXNIP-MT. Cells were transfected with luciferase vectors and miRNAs. Luciferase activity was measured after 48 h transfection using the dual-luciferase reporter kit (Promega).

### 2.8. RNA Pull-Down Assay

HCC cell lysates were incubated with the biotin labeled miR-519c-3p-WT or MT at room temperature for 2 h. Then, streptavidin-labeled magnetic beads (Invitrogen) were added and incubated for 4 h. After washed with lysis buffer, RNA was extracted and subjected to RT-qPCR analysis.

### 2.9. RNA Immunoprecipitation (RIP) Assay

RIP assay was performed using the Magna RI RIP Kit (Millipore, Billerica, MA, USA). Briefly, cells were lysed and incubated with anti-Ago2 or anti-IgG antibody at 4°C for 2 h. Then, samples were mixed with magnetic beads at 4°C for overnight. Finally, beads were washed with RIP buffer and treated with TRIzol to isolate RNA sample and then subjected to RT-qPCR analysis.

### 2.10. Detection of MAGI2-AS3, miR-519c-3p, and TXNIP Expression at Public Access Tool

The expression levels of MAGI2-AS3, miR-519c-3p, and TXNIP in HCC tissues and normal tissues were analyzed at ENCORI website. Correction of MAGI2-AS3 with miR-519c-3p or TXNIP was also analyzed at ENCORI.

### 2.11. Xenograft Model

Study protocol was approved by Affiliated Hospital of Youjiang Medical University for Nationalities. BALB/c nude mice were injected with HCC cells with or without MAGI2-AS3 overexpression. Tumor width and length were measured every 7 days to calculate tumor volume with the formula: (width^2^ × length)/2. 4 weeks after injection; mice were killed to obtain tumor tissues and then weighted to record tumor weight.

### 2.12. Statistical Analysis

Data were analyzed with SPSS (IBM, Armonk, NY, USA) and presented as mean ± SD. Differences were analyzed with Student's *t*-test and one-way ANOVA. Expression correlations were analyzed using Pearson's correlation method. *P* < 0.05 was considered as statistically significant.

## 3. Results

### 3.1. MAGI2-AS3 Was Decreased Expression in HCC

**Figure 1:**
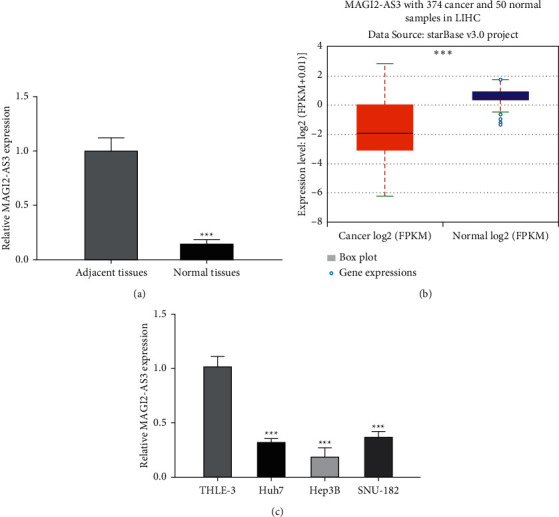
MAGI2-AS3 expression in HCC tissues and cell lines. (a) MAGI2-AS3 expression in HCC tissues and normal tissues analyzed by RT-qPCR. (b) MAGI2-AS3 expression in HCC tissues and normal tissues analyzed at ENCORI. (c) MAGI2-AS3 expression in HCC cells and normal cells analyzed by RT-qPCR. HCC, hepatocellular carcinoma; MAGI2-AS3, MAGI2 antisense RNA 3; RT-qPCR, real-time quantitative PCR.

At first, RT-qPCR analysis showed MAGI2-AS3 expression was remarkedly decreased in HCC tissues compared with adjacent normal tissues (Figure 1(a)). In addition, we analyzed the MAGI2-AS3 in ENCORI and found the MAGI2-AS3 level was also decreased in tumor tissues compared with normal tissues (Figure 1(b)). Moreover, as expected, lower MAGI2-AS3 expression was observed in HCC cells compared with normal cell (Figure 1(c)). Hep3B with the lowest MAGI2-AS3 levels among the investigated HCC cells was selected for following analyses.

### 3.2. MAGI2-AS3 Inhibits HCC Cell Proliferation In Vitro

**Figure 2:**
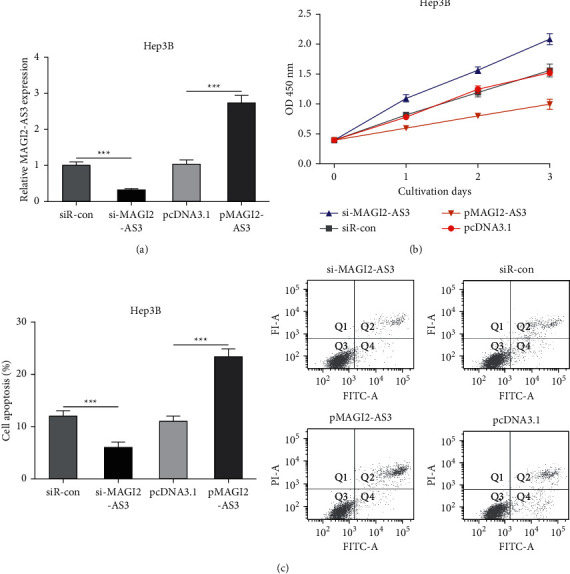
MAGI2-AS3 regulating HCC tumor progression in vitro. (a) MAGI2-AS3 expression in HCC cells with pMAGI2-AS3 or si-MAGI2-AS3 transfection. (b) Cell viability of HCC cells with pMAGI2-AS3 or si-MAGI2-AS3 transfection. (c) Cell apoptosis rate of HCC cells with pMAGI2-AS3 or si-MAGI2-AS3 transfection. HCC, hepatocellular carcinoma; MAGI2-AS3, MAGI2 antisense RNA 3; RT-qPCR, real-time quantitative PCR; si-MAGI2-AS3, small interfering RNA against MAGI2-AS3; siR-con, negative control siRNA.

To explore the roles of MAGI2-AS3 on HCC, we performed gain and loss-of-function experiments on HCC cells. Transfection efficiency was validated by RT-qPCR (Figure 2(a)). CCK-8 assay revealed that MAGI2-AS3 overexpression remarkedly suppresses, while MAGI2-AS3 knockdown significantly stimulates cell proliferation in HCC cells compared with the negative controls (Figure 2(b)). Furthermore, the results of flow cytometry showed the cell apoptosis rate in the MAGI2-AS3 overexpressed group is higher than that in the pcDNA3.1 group (Figure 2(c)). Meanwhile, MAGI2-AS3 knockdown significantly repressed cell apoptosis compared with siR-con (Figure 2(c)).

### 3.3. MAGI2-AS3 Suppresses HCC Tumorigenesis In Vivo

**Figure 3:**
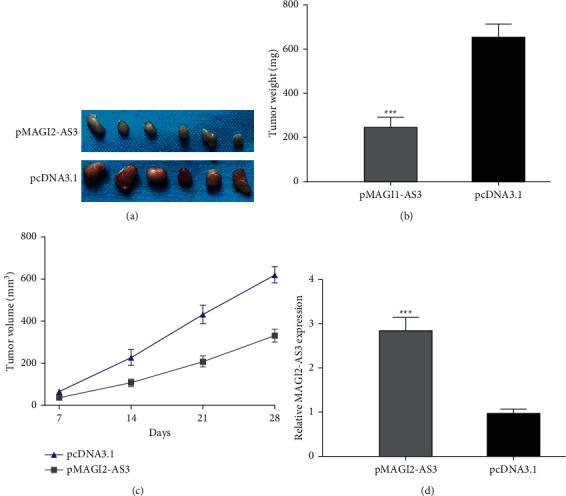
MAGI2-AS3 regulating HCC tumor progression in vivo. (a) Images of the tumor tissues transfected with pMAGI2-AS3 or pcNDA3.1. (b) Tumor size of the tumor tissues transfected with pMAGI2-AS3 or pcNDA3.1. (c) Tumor weight of the tumor tissues transfected with pMAGI2-AS3 or pcNDA3.1. (d) MAGI2-AS3 expression in tumor tissues transfected with pMAGI2-AS3 or pcNDA3.1. HCC, hepatocellular carcinoma; MAGI2-AS3, MAGI2 antisense RNA 3; RT-qPCR, real-time quantitative PCR.

Subsequently, we tested the roles of MAGI2-AS3 on HCC tumor growth in vivo. As displayed in Figure 3(a) and 3(b), tumors volume obtained from MAGI2-AS3 overexpressed groups was lower than those from control groups. Consistently, tumor weight was significantly reduced by MAGI2-AS3 overexpression (Figure 3(c)). Then, we detected MAGI2-AS3 expression levels and showed MAGI2-AS3 was significantly elevated by pMAGI2-AS3 in tumors (Figure 3(d)).

### 3.4. miR-519c-3p Was a Target for MAGI2-AS3

**Figure 4:**
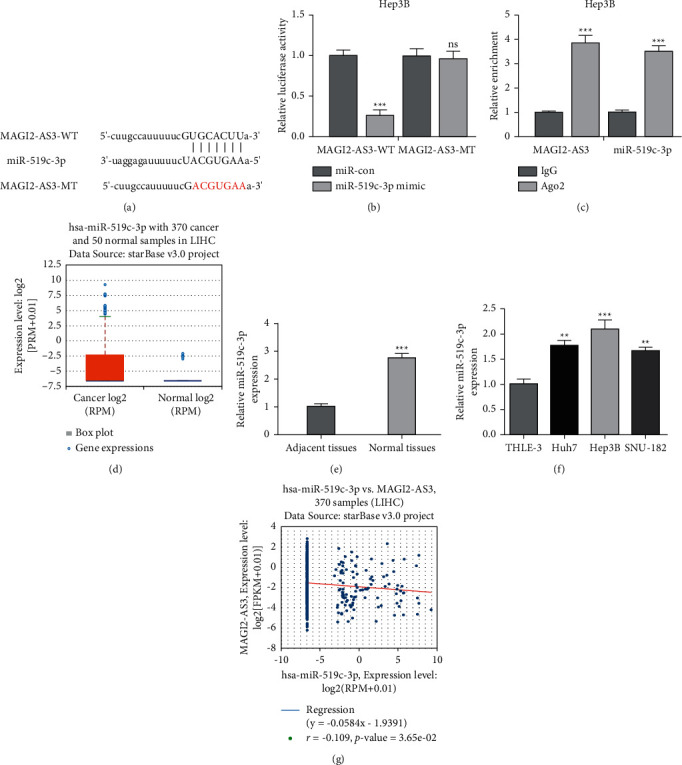
MAGI2-AS3 directly interacts with miR-519c-3p. (a) Binding region between MAGI2-AS3 and miR-519c-3p predicted by ENCORI. (b) Luciferase assay showing miR-519c-3p mimic decreased luciferase activity of MAGI2-AS3-WT but not MAGI2-AS3-MT. (c) RIP assay showing the coenrichment of MAGI2-AS3 and miR-519c-3p. (d) MAGI2-AS3 expression in HCC tissues and normal tissues analyzed at ENCORI. (e) MAGI2-AS3 expression in HCC tissues and normal tissues analyzed by RT-qPCR. (f) MAGI2-AS3 expression in HCC cells and normal cells analyzed by RT-qPCR. (g) Correlation of MAGI2-AS3 and miR-519c-3p analyzed at ENCORI. HCC, hepatocellular carcinoma; MAGI2-AS3, MAGI2 antisense RNA 3; RT-qPCR, real-time quantitative PCR; WT, wild type; MT, mutant; RIP, RNA immunoprecipitation; miR-519c-3p, microRNA-519c-3p; miR-con, negative control miRNA.

To explore the miRNA target of MAGI2-AS3 in HCC, ENCORI was performed, and the prediction results showed MAGI2-AS3 interacts with miR-519c-3p (Figure 4(a)). Luciferase assay showed miR-519c-3p mimic can decrease luciferase activity of cells transfected with MAGI2-AS3-WT but not MAGI2-AS3-MT (Figure 4(b)). Additionally, the RIP assay showed MAGI2-AS3 and miR-519c-3p were coenriched (Figure 4(c)). Moreover, we detected miR-519c-3p levels in HCC and showed miR-519c-3p expression was higher in HCC tissues and cells (Figures 4(d)–4(f)). Furthermore, we showed MAGI2-AS3 has a negative correction relationship with miR-519c-3p in HCC tissues (Figure 4(g)).

### 3.5. MAGI2-AS3 Regulates HCC Cell Proliferation by Regulating miR-519c-3p

**Figure 5:**
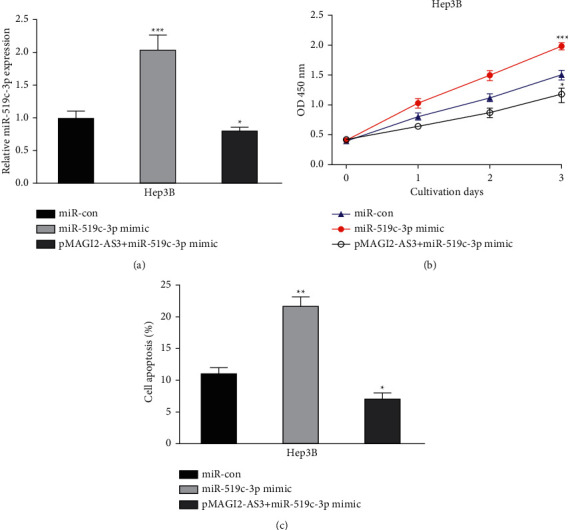
miR-519c-3p mimic restores the effects of MAGI2-AS3 on HCC cells. (a) MAGI2-AS3 expression in HCC cells with pMAGI2-AS3+miR-519c-3p mimic, miR-519c-3p mimic, or miR-con transfection. (b) Cell viability of HCC cells with pMAGI2-AS3+miR-519c-3p mimic, miR-519c-3p mimic, or miR-con transfection. (c) Cell apoptosis rate of HCC cells with pMAGI2-AS3+miR-519c-3p mimic, miR-519c-3p mimic, or miR-con transfection. HCC, hepatocellular carcinoma; MAGI2-AS3, MAGI2 antisense RNA 3; RT-qPCR, real-time quantitative PCR; miR-519c-3p, microRNA-519c-3p; miR-con, negative control miRNA.

To explore whether the roles of MAGI2-AS3 are mediated by miR-519c-3p, we transfected miR-519c-3p mimic into the MAGI2-AS3 overexpressed HCC cells. RT-qPCR showed levels of the miR-519c-3p in pMAGI2-AS3 transfected group were significantly lower than the cotransfection of the pMAGI2-AS3 and miR-519c-3p mimic group or pcDNA3.1 group (Figure 5(a)). CCK-8 assay, transwell invasion assay, and flow cytometry assay showed cotransfection of miR-519c-3p mimic increased cell proliferation and invasion and decreased cell apoptosis compared with the pMAGI2-AS3 group (Figures 5(b) and 5(c)).

### 3.6. MAGI2-AS3 Regulates TXNIP Expression via Sponging miR-519c-3p

**Figure 6:**
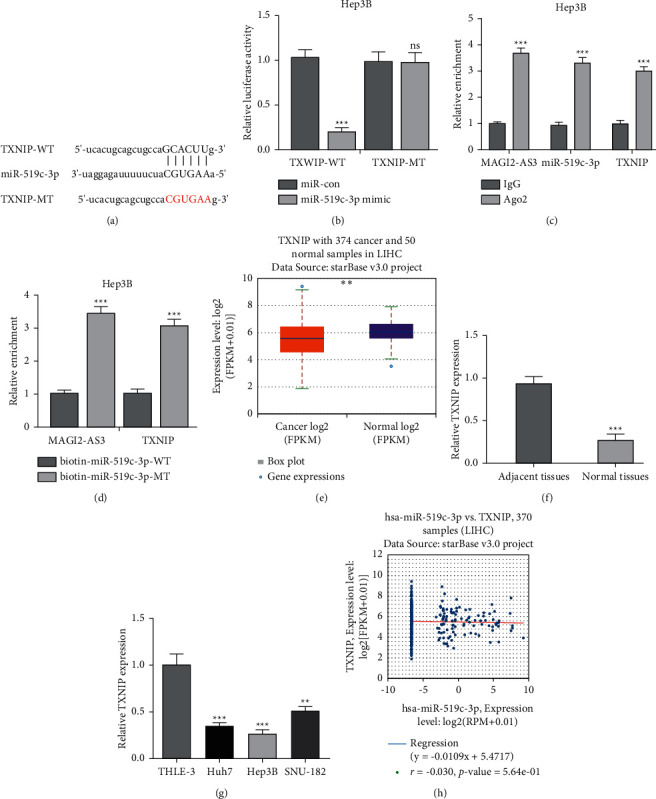
TXNIP was direct target of miR-519c-3p. (a) Binding region between TXNIP and miR-519c-3p predicted by TargetScan. (b) Luciferase assay showing miR-519c-3p mimic decreased luciferase activity of TXNIP-WT but not TXNIP-MT. (c) RIP assay showed the coenrichment of MAGI2-AS3, TXNIP, and miR-519c-3p. (d) MAGI2-AS3 and TXNIP expression in complex pulled down by biotin-miR-519c-3p-WT or biotin-miR-519c-3p-MT. (e) MAGI2-AS3 expression in HCC tissues and normal tissues analyzed at ENCORI. (f) MAGI2-AS3 expression in HCC tissues and normal tissues analyzed by RT-qPCR. (g) MAGI2-AS3 expression in HCC cells and normal cells analyzed by RT-qPCR. (h) Correlation of MAGI2-AS3 and miR-519c-3p analyzed at ENCORI. HCC, hepatocellular carcinoma; MAGI2-AS3, MAGI2 antisense RNA 3; RT-qPCR, real-time quantitative PCR; WT, wild type; MT, mutant; RIP, RNA immunoprecipitation; miR-519c-3p, microRNA-519c-3p; miR-con, negative control miRNA; TXNIP, thioredoxin interacting protein.

Next, TargetScan revealed TXNIP was a potential target of miR-519c-3p (Figure 6(a)). Luciferase reporter assay further demonstrated that miR-519c-3p overexpression decreased luciferase activity of TXNIP-WT construct, while it did not affect the activity of cells transfected with TXNIP-MT construct (Figure 6(b)). RIP and RNA pull-down assay revealed that MAGI2-AS3 and TXNIP could interact with miR-519c-3p (Figures 6(c) and 6(d)). RT-qPCR showed TXNIP was increased expression in HCC tissues and cells (Figures 6(e)–6(g)). Pearson's correlation method showed MAGI2-AS3 was positively correlated with TXNIP (Figure 6(h)).

### 3.7. MAGI2-AS3 Exerts Its Function by Regulating TXNIP in HCC

**Figure 7:**
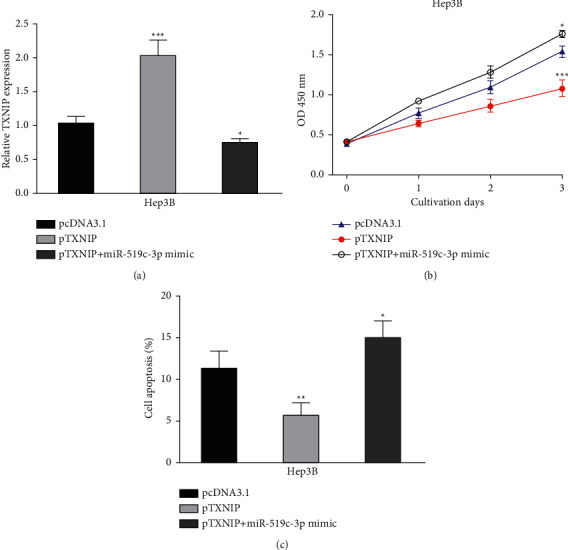
MAGI2-AS3 affects HCC behaviors through regulating TXNIP. (a) TXNIP expression in HCC cells with pTXNIP + miR-519c-3p mimic, pTXNIP, or pcDNA3.1 transfection. (b) Cell viability of HCC cells with pTXNIP + miR-519c-3p mimic, pTXNIP, or pcDNA3.1 transfection. (c) Cell apoptosis rate of HCC cells with pTXNIP + miR-519c-3p mimic, pTXNIP, or pcDNA3.1 transfection. HCC, hepatocellular carcinoma; MAGI2-AS3, MAGI2 antisense RNA 3; RT-qPCR, real-time quantitative PCR; miR-519c-3p, microRNA-519c-3p; miR-con, negative control miRNA; TXNIP, thioredoxin interacting protein.

Based on these observations, we hypothesized that MAGI2-AS3 may affect HCC progression via miR-519c-3p/TXNIP axis. We then overexpressed TXNIP in miR-519c-3p overexpressed in HCC cells. As shown in Figure 7(a), the TXNIP level in the pTXNIP group was significantly higher than that in the pcDNA3.1 or pTXNIP and miR-519c-3p mimic group. CCK-8 assay indicated that overexpression of TXNIP reversed the effects of cell proliferation induced by miR-519c-3p mimic (Figure 7(b)). Moreover, overexpression of TXNIP attenuated the effects of miR-519c-3p mimic on cell apoptosis (Figure 7(c)).

## 4. Discussion

Emerging evidence has indicated that lncRNAs are crucial mediators for the progression of HCC. For example, lncRNA taurine upregulated gene 1 was found elevated expression in HCC and promotes tumor growth and metastasis by promoting distal-less homeobox 2 expression via sponging miR-216b-5p [10]. lncRNA DLGAP1-AS1 revealed upregulated expression in HCC and promoted tumorigenesis through activating the Wnt/*β*-catenin pathway [11]. In this study, we revealed that MAGI2-AS3 was decreased expression in HCC by analyzing tissue samples, cells, and online database. Moreover, we revealed that MAGI2-AS3 could inhibit HCC cell proliferation in vitro using gain and loss-of-function experiments. In addition, we showed that MAGI2-AS3 could inhibit HCC tumor growth in vivo through gain-of-function experiment. These results indicated that MAGI2-AS3 may function as tumor suppressive role in HCC.

To date, the numbers of identified lncRNAs were gradually increasing, but the mechanisms related to their roles in carcinogenesis are largely elusive. Hence, we aimed to discover the potential mechanisms of MAGI2-AS3 in HCC using the ceRNA theory. miR-3163, miR-141, and miR-200a were previously identified as miRNA targets for MAGI2-AS3 in cancers [7, 12]. Here, we predicted and validated that miR-519c-3p was a possible target for MAGI2-AS3 using bioinformatic analysis, luciferase activity assay, and RIP assay. After that, we searched previous literature regarding the roles of miR-519c-3p in cancer and found miR-519c-3p is a newly identified miRNA with very little studies to report its functions. Therefore, considering the roles of miR-519c-3p in cancer remain largely unknown, we explored whether miR-519c-3p was a functional target for MAGI2-AS3. Our study demonstrated that the overexpression of miR-519c-3p could partially overwrite the functions of MAGI2-AS3 in HCC. These results suggested a tumor promoting role of miR-519-3p in HCC, which is in consistent with the previous study [9]. Taken together, our study combined with the previous study validated the importance of miR-519c-3p in HCC.

TXNIP, a protein induced by vitamin D3, is originally named as vitamin D3 upregulated protein 1 [13]. TXNIP was revealed decreased expression in renal cell carcinoma, and its low expression was associated with advance tumor stages and poorer overall survival [14]. In addition, TXNIP expression revealed could be induced by MondoA and to inhibit cervical cancer cell proliferation, migration, and invasion [15]. In our study, we discovered that TXNIP was a target for miR-519c-3p and MAGI2-AS3 that regulates TXNIP expression via sponging miR-519c-3p. Rescue experiments further confirmed that MAGI2-AS3 regulates HCC progression via the miR-519c-3p/TXNIP axis. A very recent study performed single-cell RNA-seq of colorectal cancer samples and showed the low activity of the MondoA-TXNIP axis is associated with the intratumoral Tregs content [16]. Also, it should be noted that suppressing the activity of MondoA-TXNIP can facilitate Th17 inflammation and stimulate CD8^+^ T cell exhaustion, indicating the roles of TXNIP in regulating tumor microenvironment [16]. As we identified that TXNIP was a target of MAGI2-AS3 and results in the reduced expression in of TXNIP in HCC and impaired cancer progression, we suspected that altered MAGI2-AS3 expression may also cause changes in the immune cell populations in the microenvironment of HCC patients. It will be interesting to further analyze the different types and proportion of immune cells after knockdown of overexpressing MAGI2-AS3 in HCC to dig out the deep mechanisms by which MAGI2-AS3 affects HCC progression.

Our previous study highlighted that MAGI2-AS3 was an epigenetic regulator to regulate the overall H3K4me2 demethylation level and affect the tumorigenesis and progression of HCC [6]. In this present study, we provide new evidence that MAGI2-AS3 could affect HCC progression via a new ceRNA mechanism, which strengthen our previous study and also new insights into the functions of MAGI2-AS3 in HCC.

## 5. Conclusion

In summary, we revealed that MAGI2-AS3 was decreased expression in HCC, and it can inhibit HCC progression via the miR-519c-3p/TXNIP axis, which provided insights that MAGI2-AS3 may be the effective target for HCC treatment.

## Data Availability

The data used to support the findings of this study are available from corresponding author upon request.
